# Nicotine dependence as a risk factor for upper aerodigestive tract (UADT) cancers: A mediation analysis

**DOI:** 10.1371/journal.pone.0237723

**Published:** 2020-08-28

**Authors:** Sera Thomas, June C. Carroll, M. Catherine Brown, Zhou Chen, Maryam Mirshams, Devalben Patel, Kevin Boyd, Andrew Pierre, David P. Goldstein, Meredith E. Giuliani, Wei Xu, Lawson Eng, Elham Khodayari Moez, Geoffrey Liu, Rayjean J. Hung

**Affiliations:** 1 Lunenfeld-Tanenbaum Research Institute, Sinai Health System, Toronto, Ontario, Canada; 2 Department of Family Medicine, Sinai Health System; Toronto, Ontario, Canada; 3 Department of Family & Community Medicine, University of Toronto, Toronto, Ontario, Canada; 4 Ontario Cancer Institute, Princess Margaret Cancer Center, University Health Network, Toronto, Ontario, Canada; 5 Department of Surgery, University of Toronto, Toronto, Ontario, Canada; 6 Department of Otolaryngology-Head & Neck Surgery, University of Toronto, Toronto, Ontario, Canada; 7 Department of Radiation Oncology, University of Toronto, Toronto, Ontario, Canada; 8 Dalla Lana School of Public Health, University of Toronto, Toronto, Ontario, Canada; National Health Research Institutes, TAIWAN

## Abstract

**Purpose:**

This study investigated nicotine dependence as an independent risk factor for upper aerodigestive tract (UADT) cancers, including lung and head and neck cancers (HNC). The study aimed to isolate the direct effect of nicotine dependence, independent of tobacco smoking.

**Methods:**

A case-control study with a total of 4957 participants was conducted in Ontario, Canada, of which 2964 categorized as either current or former smokers were used in the analysis. Nicotine dependence of ever-smokers (2360 UADT cases and 604 controls) was measured using the Fagerström Test for Nicotine Dependence. Using mediation analyses and adjusted logistic regression models, we decomposed the direct effect of nicotine dependence and the mediated effect of smoking duration to quantify the risks of lung and HNC. The role of human papillomavirus (HPV) and cancer subtypes were assessed.

**Results:**

Most individual nicotine dependence behaviours showed positive associations with lung cancer with approximately 1.8 to 3.5-fold risk increase, and to lesser extent with 1.4 to 2.3-fold risk for HNC. Nicotine dependence is partially accountable for increased risks of lung cancer (OR = 1.20, 95%CI = 1.13–1.28) and HNC (1.12, 95%CI = 1.04–1.19). Nicotine dependence had a greater effect on the risk of HPV-negative oropharyngeal cancer (OR = 3.06, 95%CI = 1.65–5.66) in comparison to HPV-positive oropharyngeal cancer (OR = 1.05, 95%CI = 0.67–1.65). The direct effects of nicotine dependence remained significant after accounting for cumulative tobacco exposures.

**Conclusion:**

Nicotine dependence increases the risks of lung and HNC cancers after accounting for tobacco smoking, suggesting potential toxic effects of nicotine. These results are informative for the safety consideration of nicotine exposures.

## Introduction

Upper aerodigestive tract (UADT) cancers, including lung and head and neck cancers (HNC), represent a substantial public health burden worldwide. Lung cancer accounts for 1.6 million deaths globally per year as the leading cause of cancer mortality [1]. Likewise, HNC contributes to an economic burden of $3.64 billion in direct medical costs in the United States alone [2,3]. Nicotine, the active ingredient in tobacco, is the primary contributing factor for dependence and continual tobacco use. Although nicotine is not considered carcinogenic, studies have shown that nicotine dependence is associated with increased risk of UADT cancer [4–8]. At the molecular level, nicotine stimulates several processes that contribute to cancer development such as formation of tobacco-specific N-nitrosamines within the body [9–12]. *In vitro* studies have shown that nicotine has toxic effects on cell signalling pathways, may promote cellular proliferation and endothelial cell migration [9,12]. It may enhance angiogenic growth factors and has been associated with decreased tumor suppression [9,12].

Our study objective was to examine the association between nicotine dependence and the risk of UADT cancers (lung and HNC cancers), based on the Fagerström Dependence Test. In particular, we aimed to quantify the effect of nicotine dependence on UADT cancer risk and parse out the component that is mediated through smoking duration and the direct effect of nicotine dependence, independent of smoking duration [7,8,13–15]. This is the first study that investigates the direct effect of nicotine dependence for both lung cancer and HNC in parallel.

## Materials and methods

### Study design

The research study has been approved by the Mount Sinai Hospital Research Ethics Board (08-0191-E) and the University Health Network Research Ethics Board to conduct human subject research. Written informed consent was obtained from every participating human participant prior to study involvement. The Mount Sinai Hospital-Princess Margaret Multicancer study (MSH-PMH) was conducted at Sinai Health System and Princess Margaret Cancer Centre, two adjacent academic health care institutions in the Greater Toronto Area, Canada, between October 2008 and December 2014. The eligible study population were residents of the province of Ontario aged 18 years or older. Cases were patients diagnosed with lung or head and neck cancer, collectively termed as upper aero-digestive tract (UADT) cancer. Cancer patients were identified through surgical, medical, and radiation departments; and outpatient clinic visit schedules and had confirmed histological diagnosis. Eligible cancer patients were approached during their clinic visit by study personnel. Cancer diagnosis was classified in accordance with the International Classification for Diseases ICD-10 (lung cancer coded C33-34 and HNC coded C01-11, C13-14, and C32), in addition to ICD-O-3 for morphological classification [16,17].

**Fig 1:**
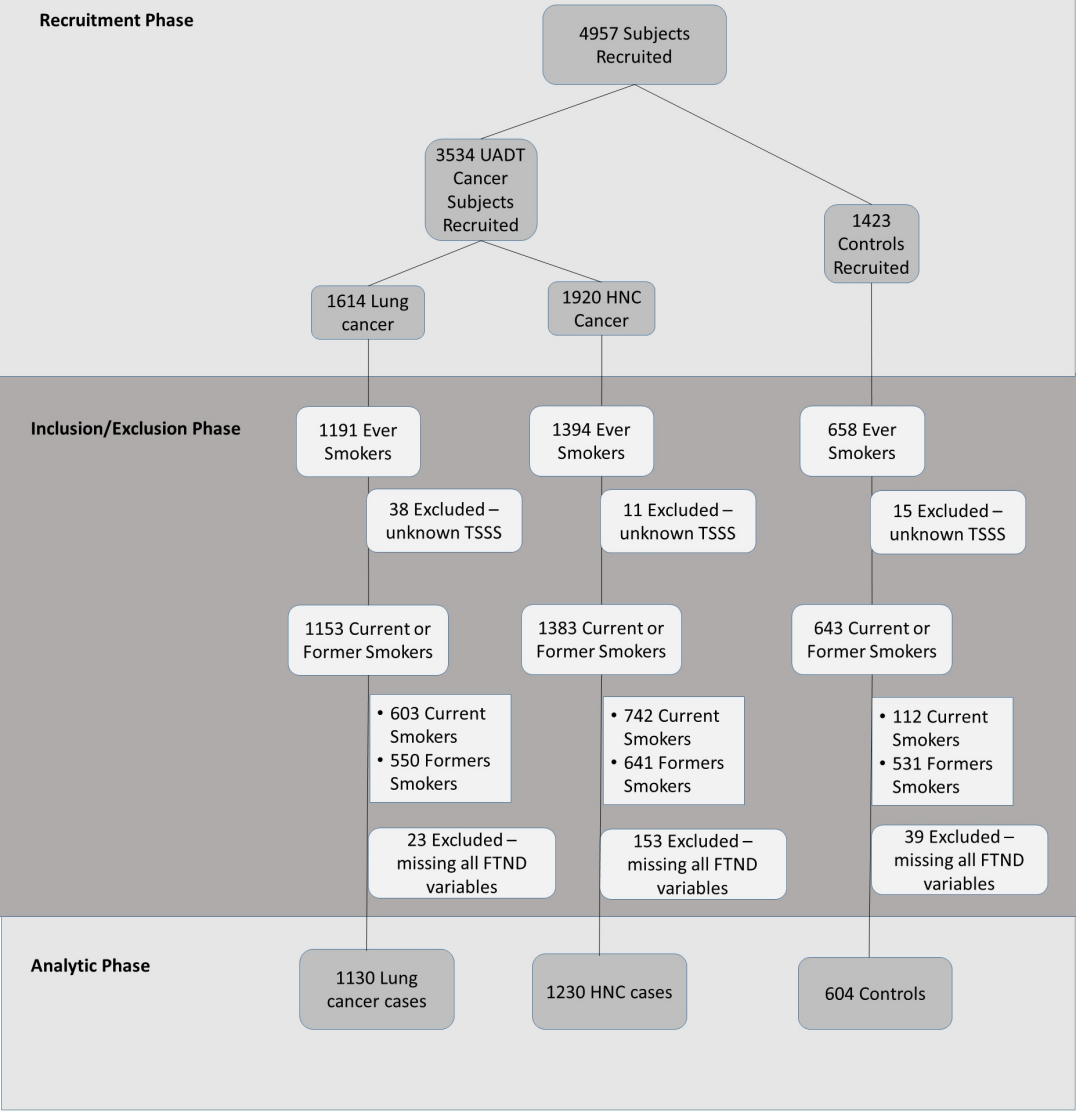
Participant data flow chart. *Recruitment phase*: A total of 4957 subjects were recruited which consisted of 3534 Upper aerodigestive tract (UADT) cancer cases and 1423 healthy controls. It included 1614 lung cancer cases and 1920 Head and Neck cancer (HNC) cases. *Inclusion/Exclusion phase*: Only current and former smokers were used in the study analysis. A total of 1153 lung cancer cases, 1382 HNC cases, and 643 controls met these criteria. Subjects who missed all FTND variables were excluded from the analysis. *Analytic phase*: 1130 lung cancer cases, 1230 HNC cases, and 604 controls were included in the statistical analysis. *TSSS = time since stopped smoking; FTND = Fagerström Test for Nicotine Dependence*.

The controls were randomly selected from residents in the same geographical regions as the cases, and registered in the databases of family medicine clinics who did not have personal history of UADT cancers, nor any type of cancer at the time of recruitment. The controls were recruited using the Dillman approach with an initial mailed introductory letter, followed by a phone contact by the study personnel to provide clarifications about the study procedures [18]. All study participants, including both cases and controls, were provided opportunities to discuss and clarify the study objectives and procedures before recruitment. In addition to the residence area, controls were frequency matched with UADT cases on age (± 5 years). Due to the large difference in the distribution by sex between lung and HNC cases, we opted to conduct frequency matching on sex with lung cancer cases. A total of 4957 participants were recruited, including 1614 lung cancer cases, 1920 HNC cases, and 1423 controls (Fig 1 and S1 Table).

The study questionnaire consisted of a list of the individual’s lifestyle factors (such as alcohol and tobacco consumption, etc.) and environmental factors (such as asbestos exposure, second hand smoke exposure, etc.), as well as personal medical and family history of cancer (S1 File). The Fagerström Test for Nicotine Dependence (FTND) consisted of six dependence variables used to quantify nicotine dependence (S2 Table) [19]. The FTND is a validated tool for measuring nicotine [19]. It is often used for assessment of dependence in relation to smoking cessation strategies such as nicotine replacement therapies. Former smokers, those who quit smoking at least two years ago, completed the Fagerström test based on their period of time when they were smoking. Smoking duration was defined as the lifetime cumulative smoking years, excluding years stopped smoking.

### Statistical analysis

Since the primary research question of our study was related to nicotine addiction, this analysis included ever smokers only, which was defined as those who smoked at least 100 cigarettes in their lifetime. The analysis included only current and former smokers which equated to 1130 lung cancer cases, 1230 HNC cases, and 604 controls (Fig 1). Participants who did not respond to any FTND questions or if their smoking status (as current versus former) was indistinguishable were excluded from the analysis (Fig 1). We conducted separate analyses for HNC and lung cancer to provide cancer-specific effect estimates. We applied multivariable logistic regression to assess the association based on adjusted odds ratio (OR) and 95% confidence intervals (95%CI). Models were adjusted by age, sex, ethnicity, education, smoking status, as well as age of start smoking and family history of UADT cancer as potential confounders [2,7], [20–22]. Missing covariate data was imputed as the median of the control group for continuous variables, and for dichotomous variables, it is assumed to have 50% of the categorical levels. In addition, the HNC analysis was adjusted for alcohol consumption, measured as the average daily grams of alcohol for all alcohol types [23,24]. This was based on standardized unit conversion of alcohol consumption in accordance with the International Agency for Research on Cancer (IARC) (http://cancer-code-europe.iarc.fr/).

The primary indicator of nicotine dependence was the total Fagerström score. Each addiction variable (S2 Table) is given a score, and the sum of the individual scores equated the total Fagerström score. Only those completed FTND questionnaire with all six items answered were included in the mediation analysis of total Fagerström score (S2 Table). In addition to the total score, we investigated associations with each of the six Fagerström nicotine addiction variables individually to assess if there was a dominant factor driving the association. This itemized analysis included participants who responded to the individual Fagerström variable.

To assess whether there were potential differential associations by disease subtype, we analyzed major lung cancer histological subgroups separately as adenocarcinoma, squamous cell carcinoma and small cell lung cancer, and three HNC subsites were analyzed separately as larynx/hypopharynx, oropharynx, and oral cavity/lip. Given the known etiological role of human papillomavirus (HPV) in HNC, specifically oropharyngeal cancer (OPC), we also assessed the association between nicotine dependence and HNC risk by HPV status (available from 524 HNC patients). The HPV positivity was determined by p16 overexpression by immunohistochemistry (IHC) as the standard. For the subset where the IHC staining for p16 was not available (22%), HPV status was determined by either HPV DNA detection or serological antibodies (such as HPV16 E16) [25].

#### Mediation analysis

**Fig 2:**
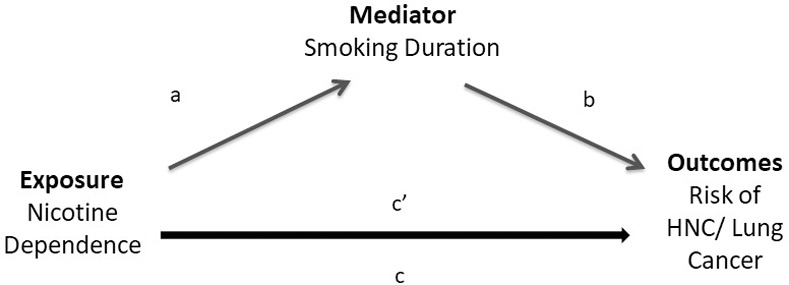
The decomposition of effects for the relationship between nicotine dependence and the risk of Head and Neck Cancer (HNC) and lung cancer, mediated through smoking duration. The total effect, denoted by ***c*,** is the logistic regression coefficient of nicotine dependence on the outcomes (risk of HNC and lung cancer) without controlling for the mediator. The direct effect, denoted by **c’**, is the effect coefficient of nicotine dependence in a logistic regression with nicotine dependence, and the mediator on the outcomes. ***a*** denotes the effect coefficient of nicotine dependence on smoking duration. ***b*** denotes the effect coefficient of smoking duration on the outcomes, in the model with both the exposure and mediator. The indirect effect, is the component of the total effect that is mediated through smoking duration (***c-c’***) in the relationship between nicotine dependence and the outcomes; it is denoted by a*b [26–28,30].

To evaluate the effect of nicotine dependence mediated through tobacco smoking on the risk of HNC and lung cancer, we conducted mediation analysis based on methods described by Vanderweele (Fig 2) [26–28]. In accordance with the counterfactual framework, we decomposed the total effect into estimates of the direct effect and the indirect effect (the mediated effect of smoking duration) on risk of HNC and lung cancer, with their associated 95% confidence intervals [26–30]. The total Fagerström score was analyzed as a continuous variable, and each item was analyzed as an ordinal variable, depending on the nature of the variable. For the HPV-related analysis, we also analyzed the modified Fagerström index based on item 1 (Time to First Cigarette (TTFC)) and item 4 (smoking intensity), as it was previously shown that the Fagerström index can reliably be reduced to these two variables to preserve statistical power when a smaller sample size or limited information is available [7,19]. Smoking duration was selected as the primary mediator, as smoking intensity (and therefore packyears) is part of the nicotine addiction measurement. To account for additional tobacco exposures not captured by smoking duration, we conducted a sensitivity analysis using packyears as the mediator and the Fagerström index without smoking intensity (item four).

The proportion mediated was estimated as OR_d_ × (OR_i_− 1) / (OR_d_ × OR_i_ − 1) where OR_i_ is the indirect effect odds ratio and OR_d_ is the direct effect odds ratio [28,31]. We included an exposure-mediator interaction in the assessment of the direct and indirect effects, which minimizes potential bias [26,27]. Specifically, the statistical coding incorporates this interaction term in the mediation model. All analyses were conducted using SAS (version 9.4; SAS Institute, Cary, NC).

Our parent study has the power to detect minimum odds ratio of 1.19 for lung cancer and 1.25 for head and neck cancers based on 5% type 1 error rate. Specifically, for this analysis, the sample sizes of the main analysis for lung cancer range from 1130 cases-604 controls to 863 casese-480 controls, which would allow us to detect a minimum direct effect odds ratio of 1.16 to 1.19 based on 5% type 1 error rate. For head and neck cancers, we can detect a minimum direct effect odds ratio of 1.20 to 1.22 based on 654 to 425 cases and 480 controls with either total or modified Fagerström scores. The minimum detectable effect sizes aligned with the expected effect sizes reported in the previous literature and are considered reasonable.

## Results

The demographic characteristics for the parent study population are summarized in S1 Table. The age distributions were comparable between patients with lung cancer, HNC and in controls. There were more males among the HNC patients compared to lung cancer cases or controls (77% versus 52% and 51% respectively) (S1 Table). The majority of the study population were of European descendant (83%). As expected, the percentage of smokers was higher in both cancer groups compared to controls (82% for lung cancer, 75% for HNC, and 47% for controls). The demographic characteristics of current and former smokers included in this analysis are summarized in Table 1. The mean age for lung cancer and HNC cases were 66 and 62 years respectively, and 62 years for controls. Overall, nicotine dependence was positively associated with the risk of both HNC and lung cancer (Table 2).

**Table 1:** Clinico-demographic characteristics of lung cancer cases, Head and Neck Cancer (HNC) cases and controls, among ever-smokers.

Characteristic	Category	Lung Cancer Case No. (%)	HNC Case No. (%)	Control No. (%)
**Total**		**1130**	**1230**	**604**
**Age in years**	Mean ± Standard Deviation	66 ± 10	62 ± 11	62 ± 12
**Sex**	Male	652 (58)	1015 (83)	319 (53)
	Female	478 (42)	215 (17)	285 (47)
**Ethnicity**	European Descent	967 (87)	1079 (88)	558 (92)
	Non-European Descent	141 (13)	151 (12)	46 (8)
**Education**	No post-secondary	570 (52)	523 (55)	100 (17)
	Any post-secondary	526 (48)	424 (45)	502 (83)
**Lung Cancer Histology**	Adenocarcinoma	639 (56)		
	Squamous Cell	246 (22)		
	Small Cell	81 (7)		
	Large Cell	42 (4)		
	Other	122 (11)		
**Head and Neck Subsites**	Oropharynx		446 (36)	
	Larynx & Hypopharynx		370 (30)	
	Oral Cavity and Lip		364 (30)	
	Other		50 (4)	
**Humanpapilloma Virus (HPV)**	With known HPV		524	
**Status**	Status			
	HPV-Positive		327 (62)	
	HPV-Negative		197 (38)	
**Family History of Lung Cancer**	Yes	192 (17)		67 (11)
	No	921 (83)		532 (89)
**Family History of Head and Neck Cancer**	Yes		64 (5)	11 (2)
	No		1166 (95)	588(98)
**Smoking Status**	Current Smoker	587 (52)	662 (54)	104 (17)
	Former Smoker	543 (48)	568 (46)	500 (83)
**Smoking Duration**	Mean ± Standard	39 ± 13	35 ± 15	22 ± 14
	Deviation			
**Packyears**	Mean ± Standard	37 ± 21	33 ± 22	17 ± 17
	Deviation			
**Age Started Smoking**	Mean ± Standard	18 ± 5	17 ± 4	19 ± 5
	Deviation			
**Average Daily Alcohol Consumption**	Mean ± Standard		19 ± 31	13 ± 16
(grams of alcohol per day)	Deviation			

**Table 2:** The Effect of nicotine dependence on the risk of lung cancer and head and neck cancer separately, mediated through duration of smoking.

		Lung Cancer						Head and Neck Cancer					
		OR	(95%CI)	Effect Type	Estimate	(95%CI)	PM	OR	(95%CI)	Effect Type	Estimate	(95%CI)	PM
1. How soon after you wake up do you smoke your first cigarette?	After 60 min	Reference		Direct	1.59	(1.39, 1.81)	63%	Reference		Direct	1.37	(1.19, 1.58)	73%
	31–60 min	1.91	(1.35, 2.71)					1.38	(0.93, 2.06)				
	6–30 min	2.62	(1.86, 3.69)	Indirect	1.12	(1.05, 1.19)		2.23	(1.54, 3.22)	Indirect	1.06	(1.02, 1.11)	
	Within 5 min	3.40	(2.22, 5.23)					2.26	(1.41, 3.62)				
2. Do you find it difficult to refrain from smoking in places where it is forbidden?	No	Reference		Direct	1.67	(1.20, 2.34)	60%	Reference		Direct	0.90	(0.60, 1.35)	n/a
	Yes	1.78	(1.28, 2.48)	Indirect	1.31	(1.09, 1.58)		0.96	(0.65, 1.43)	Indirect	1.30	(1.06, 1.58)	
3. Which cigarette would you hate most to give up?	Any Other Time	Reference		Direct	2.18	(1.60, 2.97)	46%	Reference		Direct	1.92	(1.39, 2.66)	52%
	The first in the morning	2.08	(1.56, 2.77)	Indirect	1.31	(1.10, 1.55)		1.93	(1.40, 2.67)	Indirect	1.17	(1.04, 1.32)	
4. How many cigarettes per day do you smoke?	10 or less	Reference		Direct	1.57	(1.34, 1.84)	64%	Reference		Direct	1.06	(0.91, 1.22)	95%
	11–20	2.43	(1.78, 3.31)					1.31	(0.97, 1.76)				
	21–30	3.49	(2.37, 5.13)	Indirect	1.17	(1.09, 1.26)		1.47	(0.99, 2.18)	Indirect	1.11	(1.06, 1.17)	
	31 or more	2.33	(1.40, 3.87)					0.84	(0.50, 1.40)				
5. Do you smoke more frequently during the first hours after awakening than during the rest of the day?	No	Reference		Direct	2.03	(1.38, 2.98)	49%	Reference		Direct	1.88	(1.22, 2.88)	53%
	Yes	2.11	(1.43, 3.10)	Indirect	1.18	(0.97, 1.44)		1.88	(1.24, 2.85)	Indirect	1.10	(0.95, 1.27)	
6. Do you smoke even if you are so ill that you are in bed most of the day?	No	Reference		Direct	1.04	(0.75, 1.44)	96%	Reference		Direct	0.59	(0.41, 0.87)	n/a
	Yes	1.04	(0.76, 1.44)	Indirect	1.33	(1.11, 1.60)		0.62	(0.42, 0.90)	Indirect	1.17	(1.01, 1.36)	
**Total Fagerström Score**		1.20	(1.13, 1.27)	Direct	1.20	(1.13, 1.28)	83%	1.12	(1.05, 1.20)	Direct	1.12	(1.04, 1.19)	89%
				Indirect	1.06	(1.03, 1.09)				Indirect	1.04	(1.01, 1.06)	

Models are adjusted for age, sex, ethnicity, education, smoking status, age started smoking, and family history of lung/HNC cancer. Additionally, for the HNC model, it was also adjusted for average daily alcohol consumption (grams/day). The table demonstrates the odds ratio and 95% confidence interval (OR, 95%CI) and the mediated effects associated with each Fagerström dependence variable (Items 1–6) and for the total Fagerström score (accumulated score from the scores for each of the nicotine dependence variables). “PM” is the Proportion Mediated through smoking duration. “n/a” refers to occurrences when the proportion mediated could not be estimated when the total and indirect effects were in opposite directions.

### Lung cancer

We observed a strong association between TTFC and lung cancer risk. Individuals who smoked their first cigarette within 5 minutes after waking up were three times as likely to develop lung cancer compared to those who smoked their first cigarette after 60 minutes (OR = 3.40, 95%CI = 2.22–5.23) (Table 2). Difficultly refraining from smoking in forbidden places was associated with increased risk of lung cancer (OR = 1.78, 95%CI = 1.28–2.48) (Table 2). Most of the other dependence behaviours showed a positive association with lung cancer with a two-fold increase in risk. For example, difficulty giving up first cigarette in the morning and frequent smoking after waking up in comparison to the rest of the day, showed positive associations with risk of lung cancer (OR = 2.08, 95%CI = 1.56–2.77 and OR = 2.11, 95%CI = 1.43–3.10, respectively) (Table 2). The only exception was addiction variable related to smoking while ill in bed, where there was not a relationship between those who smoke while ill in bed and lung cancer risk.

Overall, we estimated that 83% of the effect of nicotine dependence on lung cancer risk was mediated through smoking duration (Table 2). This demonstrates that the majority of the effect of nicotine addiction on lung cancer risk was mediated through smoking duration, however there was a small proportion as the potential direct effect. Thus, nicotine dependence does play a role in lung cancer.

When considering lung cancer histology, the associations between nicotine dependence and lung cancer were strongest for squamous cell carcinoma, as expected (Table 3). While the association with adenocarcinoma was less prominent compared to squamous cell, there was still a consistent positive association with nicotine dependence. For example, individuals who reported a TTFC within 5 minutes had an increased risk of lung cancer compared to those with time to first smoke after 60 minutes with ORs of 4.11 (95%CI = 1.56–6.55), 5.55 (95%CI = 2.90–8.10), and 2.58 (95%CI = 1.62–4.13) for small cell, squamous cell carcinoma and adenocarcinoma, respectively. Similar patterns were observed for the other addiction measurement items. In terms of proportion mediated through smoking duration, the estimates were comparable between squamous cell (78%) and adenocarcinoma (86%). We observed the direct effects were slightly stronger in comparison to the indirect effects for all histological types (Table 3). Nicotine dependence was slightly less associated with adenocarcinoma compared to other histological groups: the direct effect for adenocarcinoma was 1.16 (95%CI = 1.08–1.23) versus 1.28 (95%CI = 1.16–1.40) for squamous cell carcinoma (Table 3).

**Table 3:** The Effect of nicotine dependence by lung cancer histology and head and neck cancer subsite, mediated through duration of smoking.

			Direct Effect		Indirect Effect		Total Effect		Proportion Mediated (%)
	Cases (N)	Controls (N)	Effect Estimate	(95%CI)	Effect Estimate	(95%CI)	Effect Estimate	(95%CI)	
**Lung Cancer**									
Adenocarcinoma	487	480	1.16	(1.08, 1.23)	1.06	(1.03, 1.09)	1.22	(1.14, 1.31)	86%
Squamous	190	480	1.28	(1.16, 1.40)	1.11	(1.05, 1.17)	1.41	(1.27, 1.57)	78%
**Head and Neck Cancer**									
Larynx and Hypopharynx	150	480	1.15	(1.03, 1.27)	1.07	(1.02, 1.11)	1.22	(1.10, 1.36)	87%
Oropharynx	199	480	1.10	(1.01, 1.20)	1.04	(1.01, 1.07)	1.15	(1.05, 1.25)	81%
Oral cavity and Lip	161	480	1.09	(1.00, 1.20)	1.02	(1.00, 1.05)	1.12	(1.01, 1.24)	80%

Lung cancer models are adjusted for age, gender, ethnicity, education, smoking status, age started smoking, and family history of lung cancer. HNC Cancer models are adjusted for age, gender, ethnicity, education, smoking status, age started smoking, family history of HNC cancer and average daily alcohol consumption (grams/day). The table demonstrates the decomposition of effects associated with the total Fagerström score (accumulated score from each dependence variable) separately for lung cancer histology groups and HNC subsites.

Both males and females showed a positive association among nicotine dependence and individual dependence behaviours and lung cancer risk. There were slight variations in the effects mediated in males versus females, where the proportion mediated was slightly higher for males (87%) than for females (79%) based on the overall addiction score.

### Head and neck cancer

The main associated nicotine dependence measurements for HNC risk were those related to smoking during early hours. The ORs of HNC for the dependent variables, TTFC, hate to give up the first smoke in the morning, and frequent smoking during first awake hours were 2.26 (95%CI = 1.41–3.62); 1.93 (95%CI = 1.40–2.67); and 1.88 (95%CI = 1.24–2.85) respectively (Table 2). Overall, as nicotine dependence increased based on the Fagerström scale, the risk of developing HNC cancer increased by 12% when model covariates were kept constant (Table 2). After decomposition, both indirect and direct effects were positively associated with HNC, with a significant direct effect for the total Fagerström score (OR = 1.12, 95%CI = 1.04–1.19).

For larynx and hypopharynx cases, smoking a first cigarette within five minutes after waking was significantly associated with developing HNC (OR = 3.54, 95%CI = 1.26–5.81). Similar trends were shown in oropharynx and oral cavity subsites, (OR = 3.77, 95%CI = 1.59–5.94 and OR = 4.16, 95%CI = 1.73–6.60 respectively). There were slight variations in the indirect effects across HNC subsites, with effect estimates of 1.07 (95%CI = 1.02–1.11), 1.04 (95%CI = 1.01–1.07), and 1.02 (95%CI = 1.00–1.05) for larynx and hypopharynx, oropharynx and oral cavity, respectively (Table 3). After decomposition, there were no significant differences in the direct effects of nicotine dependence across all HNC subsites (Table 3).

Of the HNC with known HPV status, 62% had positive tests and the majority of the positive tests (82%) were oropharynx. Among OPC patients, HPV positive patients were younger (mean age of 59 ± 9 (standard deviation) years *versus* vs 63 ± 11 years, p < 0.05); had shorter smoking durations (mean duration 26 ± 15 years among HPV positive vs 38 ± 13 years among HPV negative; p-value <0.05); and had lower nicotine dependence scores (mean score 3.4 ± 2.3 among HPV positive versus 4.5 ± 2.5 among HPV negative; p-value < 0.05). Nicotine dependence had a stronger direct effect on OPC risk among those who were HPV-negative compared to those who were HPV-positive, with OR of 2.15 (95%CI = 1.24–3.73) and 0.99 (95%CI = 0.41–2.39) respectively (Table 4). The total effects of nicotine dependence for OPC risk were greater in those who were HPV negative with ORs of 3.06 (95%CI = 1.65–5.66) versus 1.05 (95%CI = 0.67–1.65) for those who were HPV positive (Table 4). When examining the effect of HPV for all HNC subsites together, there was a significant direct effect of nicotine dependence for HPV-negative HNC (1.63, 95%CI = 1.01–2.64), but not for HPV-positive HNC with direct effect of 0.88 (95%CI = 0.38–2.06).

**Table 4:** The effect of HPV status on Oropharyngeal Cancer (OPC).

			Direct Effect		Indirect Effect		Total Effect	
	Cases (N)	Controls (N)	Effect Estimate	(95%CI)	Effect Estimate	(95%CI)	Effect Estimate	(95%CI)
**Modified Fagerström Score**								
HPV Positive-OPC	281	567	0.99	(0.41, 2.39)	1.15	(1.03, 1.28)	1.05	(0.67, 1.65)
HPV Negative-OPC	118	567	2.15	(1.24, 3.73)	1.42	(1.14, 1.77)	3.06	(1.65, 5.66)

Models are adjusted for age, gender, ethnicity, education, smoking status, age started smoking, and family history of HNC cancer, and average daily alcohol consumption (grams/day). The table demonstrates the direct effect of nicotine dependence by HPV status within Oropharyngeal Cancer (OPC) cases. The effects are demonstrated by the modified Fagerström (the combination of only Score 1: time to first cigarette and score 4: smoking intensity).

### Sensitivity analysis

When using smoking packyears as the mediator to combine the exposure of both smoking duration and smoking intensity, the direct effects of nicotine dependence on lung cancer risk based on the Fagerström index remained significant with OR of 1.18 (95%CI = 1.07–1.30, S3 Table). As expected, the mediated effect for packyears (1.25, 95%CI = 1.16–1.34) is greater than the mediated effect of smoking duration alone (1.06, 95%CI = 1.03–1.09) (Tables 2 and S3). Similarly, for HNC, the direct effect after accounting for tobacco packyears as the mediator remained to be significant as 1.16 (95%CI = 1.05–1.29) (as opposed to 1.12 95%CI = 1.04,1.19) (Tables S3 and 2). Therefore, the mediation analysis based on cumulative smoking exposures (packyears) did not affect the conclusions of the main analysis.

## Discussion

Based on our study of both lung and HNC cancers, we observed an association between nicotine dependence and UADT cancer risk, with slight variation by disease subtype. Overall, the proportion mediated through smoking duration was comparable across disease entities, albeit variation by individual addiction measurements. Our study suggests the effect of nicotine dependence was not entirely mediated through smoking behaviour itself, as evidenced based on the direct effect from nicotine dependence, after accounting for either tobacco duration or cumulative exposure including intensity (packyears).

Our findings are compatible with the previous study reported by Gu et al. which was focused mainly on TTFC [8]. They reported an increased risk of lung cancer with shorter TTFC, similar to our observation. In addition, Gu et al. observed the strongest association for patients with squamous cell carcinoma and weakest among those adenocarcinoma, which is consistent with our results.

Similar to previous studies, our population of HPV-positive HNC cases were younger, more likely to be male, and had less smoking and alcohol exposures [32–34]. Our results highlight the distinct etiological differences by HPV status: where among HPV-negative OPC cases, higher nicotine dependence clearly influenced the risk of HNC cancer, but not the case for HPV-positive OPC cases. Furthermore, it is previously shown that HPV-positive OPC tumors tend to have wild-type p53 and p16 overexpression [32]. In comparison, similar to other HNC subsites, HPV-negative OPC tumors tend to have p53 mutations and does not exhibit p16 over-expression [32]. These observations illustrate the molecular differences of OPC tumors by HPV status. Though our HPV testing sample size is limited by subsite, there was an observed increase of HPV-positive OPC among known p16 status tests. This is the first study that assessed the effect of nicotine dependence on HNC risk by HPV status.

Our findings are consistent with the previous HNC and lung cancer studies which focused only on select variables of nicotine dependence [7,8]. Previous *in vitro* studies on cell cultures have shown that nicotine changes cell phenotypes, contributing to epithelial-to-mesenchymal transition and enabling cancer metastases [9,10,12]. Nicotine causes a decrease in the tumor suppressor Chk2, suggesting that a possible role of nicotine exists in disrupting signalling pathways for genetic surveillance and thereby facilitating carcinogenesis [9]. Through the promotion of survivin expression, nicotine decreases apoptosis and can thus facilitate angiogenesis [12]. Another mouse model study showed that mice exposed to e-cigarette smoke caused lung cancer while exposure to e-cigarette smoke without nicotine did not induce lung cancer, and this was implicated by the damage of DNA from metabolites of nicotine nitrosation products [35]. Future research examining the toxicity of nicotine directly related to UADT cancer risk in humans is warranted.

The observation of a direct effect of nicotine dependence on UADT risk may have clinical significance, particularly since nicotine delivery devices such as electronic-cigarettes have been considered as a potential harm reduction tool, amid recent rises of vaping-related injuries [36–39]. Furthermore, nicotine replacement therapy is commonly prescribed in accordance with the Fagerström categories (high, medium, low nicotine dependence) because there is a good correlation between the Fagerström test score and serum levels of nicotine [15,40]. Our findings highlight that nicotine replacement therapy should be used as a temporizing solution to allow for the eventual discontinuation of all forms of nicotine exposure, and not as a permanent alternative to cigarette smoking.

There were several limitations to the study. First, the Fagerström test is a self-reported measure of nicotine dependence with potential misclassification, however, it is a validated and widely used measurement tool in clinical settings [19,41]. Our approach allowed for a bias-correction analytic approach, a weighted mediator regression and a non-linear equation correction to adjust for case-control study designs [27]. Second, there was potentially residual confounding of smoking variables. Smoking duration represents the optimal mediator for this study, since smoking intensity is part of nicotine dependence assessment. After addressing this by using the mediator as the cumulative exposures of both duration and intensity (packyears), this did not materialistically change the conclusion that there is a direct effect from nicotine to UADT risk. While we cannot preclude the possibility of residual confounding from other smoking topography, this mediation analysis offers a structured method to decompose direct and indirect effects, while considering the potential interaction between the mediator and exposures [26]. Finally, the power for the subgroup analysis can be limited and will need to be validated in a larger study population.

In conclusion, our study isolated the direct effect of nicotine dependence on the risk of UADT cancer, independent of smoking exposure. The differential effect of nicotine addiction by HPV status highlights the importance of different etiological factors in head and neck cancer by their HPV status. In addition to the recent report of vaping-related lung illnesses, this study is particularly relevant in the era of nicotine replacement therapy and electronic cigarettes, which has increasing usage among youth population [36–38,42].

## Supporting information

S1 File MSH-PMH Study questionnaire. (PDF)

S1 Table Clinico-demographic characteristics of lung cancer cases, Head and Neck Cancer (HNC) cases and controls based on all study participants recruited in the MSH-PMH study. (DOCX)

S2 Table Fagerström dependence variable participant response rate and scoring. (DOCX)

S3 Table The effect of nicotine dependence on the lung cancer and head and neck cancer risk, mediated through tobacco packyears. (DOCX)
