# Supplementary material for: Nicotine dependence as a risk factor for upper aerodigestive tract (UADT) cancers: A mediation analysis
Source: PLoS One. 2020 Aug 28;15(8):e0237723. doi: 10.1371/journal.pone.0237723 (PMC7454981; doi:10.1371/journal.pone.0237723)
Supplement: S1 File — (PDF) [file pone.0237723.s001.pdf]

Thank you for completing this questionnaire.

Multicancer Case-control Study

Samuel Lunenfeld Research Institute, Mount Sinai Hospital

60 Murray St., Box 18, Toronto, ON M5T 3L9 416-586-4800 ext. 8134

(Office use only) **Form collected by**:

____

MOUNT SINAI HOSPITAL
Joseph and Wolf Lebovic Health Complex
Samuel Lunenfeld Research Institute

«PatientID»
«PatientID»

Multicancer Case-Control Study

Health Questionnaire

Today's Date: ☐☐☐☐ / ☐☐ / ☐☐
Year *month* *day*

Date of birth: ☐☐☐☐ / ☐☐
Year *month*

Gender: ☐ Male ☐ Female

Version 12, 06-Feb-2012

Please answer the following questions about your health now and in the past and about some of your habits and medication use. This information will be kept strictly confidential and will only be used for medical research purposes as specified on the consent form.

PART A. General Demographic and Health Information

1. What is your current **weight?** _____lb *OR* _____kg
2. What was your **weight when you were 25 years old?** _____lb *OR* _____kg
3. What is your current **height?** _____ft _____in *OR* _____cm
4. (a) In what occupation have you worked the longest during your adult life?

   ____

   (b) What were your usual activities and duties in this occupation?

   ____

   (c) How many years have you worked in this occupation? _____ years.
5. Up until the past year, you would say your health was:

   ☐ Excellent ☐ Very Good ☐ Good ☐ Fair ☐ Poor
6. Race:

   ☐ White/Caucasian ☐ Black/African-Canadian ☐ Asian/Pacific Islander

   ☐ First Nations ☐ Latino/Hispanic ☐ Mixed, specify ______________

   ☐ Other, specify _________________
7. Highest level of education completed:

   ☐ Elementary School ☐ Some High School ☐ High School Graduate

   ☐ Vocational/Technical ☐ College/University ☐ Graduate/Professional School

   ☐ Other ______________________________
8. How many years of education have you completed (starting from elementary school)?

   _____ years
9. What is your current marital status?

   ☐ Single ☐ Separated ☐ Divorced ☐ Married or living with partner ☐ Widowed

PART I. Reproductive History [for women only]

1. At what age did your menstrual periods begin? _____ years old
2. Have you completely stopped having menstrual periods?

   ☐ No ☐ Yes → at what age? _____ years old
3. (a) Have you ever been pregnant?

   ☐ No → please go to question I.4. ☐ Yes

   (b) If yes to (a), how many times? _____ #; and (c) how many live births have you had? ______#

   (d) If yes to (a), when was your first pregnancy? _____ years old
4. (a) Have you ever used birth control pills for more than 1 year?

   ☐ No → please go to question I.5. ☐ Yes

   (b) If yes to (a), when did you first start taking it? At _____ years old;

   (c) If yes to (a), for how many years did you take them? (not counting any time that you stopped for more than 6 months) _____ years
5. Have you had an operation to remove your uterus?

   ☐ No ☐ Yes, at what age? _____ years old
6. Have you had an operation to remove both of your ovaries?

   ☐ No ☐ Yes, at what age? _____ years old

PART H. *Asbestos Exposure*

1. Have you ever been exposed to asbestos?

☐ No → go to the next page.   ☐ Yes, continue.   ☐ Don't know.

(a) If yes, how often were you exposed?

☐ Once or twice   ☐ For less than 3 months   ☐ Between 3-6 months

☐ For more than 6 months

(b) When were you exposed?

From the age of _____ years to the age of _____ years

(c) Where did the exposure happen?

☐ At home   ☐ At work; Fill in job name/type below.

☐ Both; Fill in job name/type_________________________________

(d) Was the asbestos: ☐ intact or sealed? OR/AND ☐ loose and flaky?

(e) If you worked directly with asbestos, please describe your activities (i.e. spraying, applying, cutting, removing):

____

(f) What type of asbestos were you in contact with?

☐ Serpentine/Crysotile (white)   ☐ Amphiboles (brown/off-white)

☐ Crocodite (blue)   ☐ Unknown

(g) Did you wear any personal protective gear (e.g. mask)?

☐ No   ☐ Yes, from _____ (year) to _____ (year)

(h) If yes to (g), did you wear this gear appropriately?

☐ >50% of the time   ☐ <50% of the time   ☐ Never

(i) Did you work in areas where other workers were generating asbestos dust?

☐ No   ☐ Yes

Describe: ____

PART B. *Alcohol*

1. Have you ever had a drink of alcohol **at least once per week for 1 year** or longer?

☐ No

☐ Yes → please fill in table chronologically:

(a) From Age (years)	(b) To Age (years)	(c) Beer (cans or bottles/week)	(d) Wine (6-ounce glasses/week)	(e) Spirit or hard liquor (ounces/week)
		cans/bottles	glasses	ounces
		cans/bottles	glasses	ounces
		cans/bottles	glasses	ounces
		cans/bottles	glasses	ounces

2. Do you currently drink alcohol at least once per week?

☐ No→ Stopped at _____ years old

☐ Yes

PART C. *Smoking*

1. Have you smoked more than 100 **cigarettes** in your lifetime?

☐ No →please go to question C.4.

☐ Yes

2. If you have smoked more than 100 **cigarettes** in your lifetime, when did you start smoking *regularly*? _____ years old

(a) Have you stopped smoking **cigarettes now**?

☐ No

☐ Yes → (b) When did you last smoke cigarettes? _____ / _____ *OR*
At _____ years old   *Month / Year*

3. ***Please answer the following questions based on the average situation during the period when you smoked.***

(a) On average, about how many **cigarettes** do / did you smoke each day?

_____ cigarettes per day

(b) How soon after you wake up do / did you smoke your first cigarette?

☐ after 60 minutes

☐ 31-60 minutes

☐ 6-30 minutes

☐ within 5 minutes

(c) Do / did you find it difficult to refrain from smoking in places where it is forbidden. e.g. in a hospital or on a bus?

☐ No ☐ Yes

(d) Which cigarette would you hate most to give up?

☐ The first in the morning ☐ Any other

(e) Do / did you smoke more frequently during the first hours after waking than the rest of the day?

☐ No ☐ Yes

(f) Do / did you smoke when you are so ill that you are in bed most of the day?

☐ No ☐ Yes

(g) Have you ever tried to quit smoking?

☐ No ☐ Yes → How many times? ______ times

4. (a) Have you ever smoked **cigars at least once a week for 1 year** or longer?

☐ No → please go to question C.5. ☐ Yes

(b) **If yes**, average number of cigars per week _____ ; Start date _____/_____ *OR* AGE ______ years old (month / year)

(c) Do you still smoke cigars now?

☐ No → Stop date _____ / _____ *OR* AGE _____ years old (month / year)

☐ Yes

**6. Other systems**<br>**Have you ever had or been diagnosed by a medical professional as having:**	***NO***	***DON'T KNOW***	***YES*** *(If yes, at what age?)*
Diabetes	☐	☐	☐ At _____ years old
Kidney complications due to the diabetes?	☐	☐	☐ At _____ years old
Polycythemia Vera (a specific blood disease)	☐	☐	☐ At _____ years old
AIDS / HIV positive	☐	☐	☐ At _____ years old
Tuberculosis	☐	☐	☐ At _____ years old
Malaria	☐	☐	☐ At _____ years old
Kidney problems, Specify ________	☐	☐	☐ At _____ years old
Gallstones	☐	☐	☐ At _____ years old
Cholecystitis or gall bladder problems	☐	☐	☐ At _____ years old
Eczema	☐	☐	☐ At _____ years old
Drug Allergies, Specify __________	☐	☐	☐ At _____ years old
Non-drug Allergies, Specify ______	☐	☐	☐ At _____ years old
Peripheral vascular disease (blocked arteries in your legs/poor circulation causing purple feet/hands)	☐	☐	☐ At _____ years old

7. Other infection history (serious infections that lasted for at least 1 month, OR required treatment or medication for at least 1 month)

Name of the infection/disease	Age at diagnosis	Was it treated? If YES, what was the treatment?	Duration of treatment
		☐ No ☐ Yes, specify_____________________	months
		☐ No ☐ Yes, specify_____________________	months

8. Do you have other ailments not mentioned above?

____

9. Do you have more comments to make about your health?

____

Have you ever had **regurgitation**?	☐ No → please go to question G.4.<br>☐ Don't know ↗<br>☐ Yes , At _____ years old
At its worst in your life, how often did you have regurgitation?	☐ Less than once a month ☐ At least once a month<br>☐ At least once a week<br>☐ 3-4 times a week ☐ More than 4 times a week ☐ Don't know
How often was the regurgitation at night?	☐ Less than once a month ☐ At least once a month<br>☐ At least once a week<br>☐ 3-4 times a week ☐ More than 4 times a week ☐ Don't know
How long have you had symptoms of regurgitation? _____ years	

**4. Muscle, Joints and Bones**<br>**Have you ever had or been diagnosed by a medical professional as having:**	***NO***	***DON'T KNOW***	***YES*** *(If yes, at what age?)*
Rheumatoid arthritis (This is not Osteoarthritis)	☐	☐	☐ At _____ years old
Lupus (SLE), Scleroderma, Sjogrens or Connective tissue disease	☐	☐	☐ At _____ years old
Other arthritis, Specify ___________________	☐	☐	☐ At _____ years old
Gout	☐	☐	☐ At _____ years old

**5. Brain System**<br>**Have you ever had or been diagnosed by a medical professional as having (a):**	***NO***	***DON'T KNOW***	***YES*** *(If yes, at what age?)*
Stroke	☐	☐	☐ At _____ years old
Depression	☐	☐	☐ At _____ years old
Transient ischemic attack (TIA) or mini-stroke	☐	☐	☐ At _____ years old
Alzheimer's Disease (dementia)	☐	☐	☐ At _____ years old

5. (a) Have you ever smoked a **pipe at least once a week for 1 year** or longer?

   ☐ No → please go to question C.6.

   ☐ Yes

   (b) **If yes**, how many ounces/week _____ ; Start date ____ / ____ *OR* AGE ____
   (Month / Year)

   (c) Do you still smoke a pipe now?

   ☐ No → Stop date _____ / _____ *OR* AGE _____ years old
   (month / year)

   ☐ Yes

6. **Have you ever used:**

	**Never**	**Occasionally**	**Regularly**	**If yes, age when started?**
(a) Chewing tobacco?	☐	☐	☐	_____ years
(b) Snuff?	☐	☐	☐	_____ years

7. (a) At any time in your life, have you been around **someone else's tobacco smoke at least once per week for 1 year or longer**? ☐ No ☐ Yes → please fill in table:

(b) Where have you been around someone else's tobacco smoke? (check all that apply)	(c) On average, how often were you around other people's smoke?	(d) On average, how many hours of exposure did you encounter for each day you were exposed?	(e) How many years were you exposed in total?
☐ At home?<br>☐ At work?<br>☐ At leisure? (visiting friends, bars)?	☐ Daily<br>☐ At least 4 days/week but not everyday<br>☐ 1-3 days/week<br>☐ Occasionally (less than 1 day/ week)	☐ <2 hours<br>☐ 2-4 hours<br>☐ 4-6 hours<br>☐ >6 hours	#_____ years

8. In your lifetime, you have considered your **second-hand smoke exposure** to be:

   ☐ light

   ☐ moderate

   ☐ heavy

The next question is about marijuana. Please remember that your answers will remain strictly confidential

9. Have you ever used or tried marijuana, cannabis or hashish?

☐ No, Don't know or refuse → Please go to Part D: Family History
☐ Yes, just once ↗
☐ Yes, more than once → Please fill in table below

	From Age (years)	To Age (years)	Quantity of Use	Mode (please circle)	Type (please circle)
i			# _____ per day/week/month/year (please circle)	Joints/pipes/bongs	Number/ounces/hits
ii			# _____ per day/week/month/year (please circle)	Joints/pipes/bongs	Number/ounces/hits
iii			# _____ per day/week/month/year (please circle)	Joints/pipes/bongs	Number/ounces/hits

3. Digestive System - The following questions are about your digestive system.

**Have you ever had or been diagnosed by a medical professional as having:**	***NO***	***DON'T KNOW***	***YES*** *(If yes, at what age?)*
Hiatus hernia	☐	☐	☐ At _____ years old
Jaundice	☐	☐	☐ At _____ years old
Stomach ulcer or peptic ulcer proven by a test	☐	☐	☐ At _____ years old
Hepatitis	☐	☐	☐ If yes, what types?
☐ Hep. A, At _____ years old; ☐ Hep. B, At _____ years old;<br>☐ Hep. C, At _____ years old; ☐ Other hepatitis, specify ___________, At _____year old;<br>☐ Don't know, but at _____ years old			
Barrett's esophagus	☐	☐	☐ At _____ years old
Liver disease, Specify _____________	☐	☐	☐ At _____ years old
H. Pylori infection (bacteria that causes ulcers)	☐	☐	☐ At _____ years old
Ulcerative colitis/Crohn's disease	☐	☐	☐ At _____ years old
Diseases of the pancreas other than diabetes	☐	☐	☐ At _____ years old
Gastrointestinal hemorrhage/ bleeding from the digestive system	☐	☐	☐ At _____ years old

Have you ever had **heartburn**?	☐ No → please go to next table about regurgitation.<br>☐ Don't know ↗<br>☐ Yes , At _____ years old
At its worst in your life, how often did you have heartburn?	☐ Less than once a month ☐ At least once a month<br>☐ At least once a week<br>☐ 3-4 times a week ☐ More than 4 times a week ☐ Don't know
How often was the heartburn at night?	☐ Less than once a month ☐ At least once a month<br>☐ At least once a week<br>☐ 3-4 times a week ☐ More than 4 times a week ☐ Don't know
How long have you had symptoms of heartburn? _____ years	

PART G. Other Health Conditions

The following questions are about problems you may have had in the past. For those conditions that you have ever had, please specify at what age you were first told by a medical professional about it**?**

1. Lung System – The following questions are about your lungs.

**Have you ever had or been diagnosed by a medical professional as having:**	***NO***	***DON'T KNOW***	***YES*** *(If yes, at what age?)*
Asbestosis	☐	☐	☐ At _____ years old
If yes, has it been treated?	☐ No	☐Yes	If yes, in what year?_______
Pleural plaques	☐	☐	☐ At _____ years old
If yes, has it been treated? ☐ No ☐ Yes	If it has been biopsied, what were the results? _______		
Shortness of breath climbing one flight of stairs	☐	☐	☐ At _____ years old
Pneumonia	☐	☐	☐ At _____ years old
Chronic Bronchitis/COPD/Chronic Obstructive Pulmonary Disease	☐	☐	☐ At _____ years old
Emphysema	☐	☐	☐ At _____ years old

2. Heart System – The following questions are about your heart.

	NO	***DON'T KNOW***	***YES*** *(If yes, at what age?)*
Have you ever seen a cardiologist (heart specialist)?	☐	☐	☐ At _____ years old
Have you ever had investigations or tests done for your heart?	☐	☐	☐ At _____ years old
Have you ever had a coronary artery bypass surgery?	☐	☐	☐ At _____ years old
**Have you ever been diagnosed by a medical professional as having:**			
High blood pressure (hypertension)	☐	☐	☐ At _____ years old
High cholesterol	☐	☐	☐ At _____ years old
Heart failure	☐	☐	☐ At _____ years old
Angina (chest pain from the heart)	☐	☐	☐ At _____ years old
Heart attack (myocardial infarction)	☐	☐	☐ At _____ years old
Angioplasty	☐	☐	☐ At _____ years old
Irregular heart rhythm (arrhythmia)	☐	☐	☐ At _____ years old

PART D: Family History

1. How many siblings do you have?

   _____# sisters _____ # brothers _____# half-sisters _____# half-brothers

2. How many biological children do you have? _____# daughters _____ # sons

3. Please list each blood-related mother, father, daughter, son, brother or sister that has ever had cancer.

   ☐ None

Relation	Type of cancer	Age at diagnosis	Alive? (Yes/No)	Did this person ever smoke?

4. Please list each blood-related mother, father, daughter, son, brother or sister that has ever had any **LIVER DISEASES**, for example Hepatitis A, B, C, cirrhosis, cholecystitis, gallstones, cholecyst disease or other liver diseases (liver cancer should be listed above)

   ☐ None

Relation	Type of liver disease	Age at diagnosis

5. Please list ANY of your blood-related relatives who been diagnosed with **PANCREATITIS**?

   ☐ None

Relation	Age at diagnosis	Number of bouts of pain	Number of years affected

6. Please list ANY of your blood-related relatives who been diagnosed with **DIABETES**?

☐ None

Relation	Type of diabetes (if known) (e.g. Type 1/Juvenile, Type 2/Diabetes mellitus)	Age at diagnosis

PART E. Past Cancer History

1. Please fill in the table below if **you** have ever been **diagnosed with any type of cancer** (please list all cancers including leukemia, lymphoma, and skin cancers like melanoma, basal cell or squamous cell skin cancer):

Type of Cancer (e.g. Breast, lung, etc.)	Date of Diagnosis (month and year)	Type of Treatment (e.g. surgery, radiation, chemotherapy)	Duration of treatment (months)

2. (a) Have you ever had radiation treatment for a medical problem?

☐ No

☐ Yes → please fill in table:

(b) From age	To age	For what conditions?	Which part of your body was treated?

3. **[For women only]**

(a) Have you ever had a mammogram?

☐ No → please go to ***PART F***.

☐ Yes → (b) How many mammograms? ______#

(c) How old were you when you had your first mammogram?

_____ years old

PART F. History of Aspirin Usage

1. Have you ever taken **aspirin** or **aspirin-like drugs** such as ibuprofen or Motrin **at least once per week for 6 months** or longer in any period of time during your life?

☐ No ☐ Yes → please fill in table

Check those that apply	Age started (years)	Age stopped (years)	Average amount taken per week during this period: Once a week	A few times a week, but not everyday	At least once a day	Total number of years taken
☐ Aspirin			☐	☐	☐	_____ years
			☐	☐	☐	
			☐	☐	☐	
☐ Naprosyn, Ibuprofen, Motrin or similar drugs called NSAIDS			☐	☐	☐	_____ years
			☐	☐	☐	
			☐	☐	☐	
