# Supplementary material for: Nicotine dependence as a risk factor for upper aerodigestive tract (UADT) cancers: A mediation analysis
Source: PLoS One. 2020 Aug 28;15(8):e0237723. doi: 10.1371/journal.pone.0237723 (PMC7454981; doi:10.1371/journal.pone.0237723)
Supplement: S1 Table — (DOCX) [file pone.0237723.s002.docx]

**S1 Table: Clinico-demographic characteristics of lung cancer cases, head and neck cancer (HNC) cases and controls based on all study participants recruited in the MSH-PMH study**

| **Characteristic** | **Level or Category** | **Lung Cancer Case No. (%)** | **HNC Case No. (%)** | **UADT Cancer No. (%)** | **Control No. (%)** |
| --- | --- | --- | --- | --- | --- |
| **Total** |  | **1614** | **1920** | **3534** | **1423** |
| **Age (years)** | <55 | 265 (17) | 501 (26) | 766 (22) | 403 (28) |
|  | 55-65 | 438 (27) | 658 (34) | 1096 (31) | 490 (35) |
|  | 65-75 | 617 (38) | 496 (26) | 1113 (31) | 362 (25) |
|  | >75 | 294 (18) | 265 (14) | 559 (16) | 168 (12) |
|  | *Mean Age*  *± Standard Deviation* | *66 ± 11* | *62 ± 12* |  | *61 ± 12* |
|  |  |  |  |  |  |
| **Sex** | Male | 833 (52) | 1482 (77) | 2315 (66) | 723 (51) |
|  | Female | 781 (48) | 438 (23) | 1219 (34) | 700 (49) |
|  |  |  |  |  |  |
| **Ethnicity** | European Descent | 1157 (79) | 1469 (83) | 2626 (81) | 1217 (86) |
|  | Non-European Descent | 305 (21) | 309 (17) | 614 (19) | 200 (14) |
|  | Missing | 152 | 142 | 294 | 6 |
|  |  |  |  |  |  |
| **Education** | No post-secondary | 695 (50) | 709 (51) | 1404 (51) | 186 (13) |
|  | Any post-secondary | 694 (50) | 680 (49) | 1374 (49) | 1193 (87) |
|  | Missing | 225 | 531 | 756 | 44 |
|  |  |  |  |  |  |
| **Smoking Status** | Never smokers | 262 (18) | 475 (25) | 737 (22) | 738 (53) |
|  | All Ever Smokers | 1191 (82) | 1394 (75) | 2585 (78) | 658 (47) |
|  | *Current* | *603 (42)* | *742 (40)* | *1345 (40)* | *112 (8)* |
|  | *Former* | *550 (38)* | *641 (34)* | *1191 (36)* | *531 (38)* |
|  | *Smokers, NOS* | *38 (3)* | *11 (1)* | *49 (1)* | *15 (1)* |
|  | Missing | 161 | 51 | 212 | 27 |
|  | | | | |  |
| **Histology** | Adenocarcinoma | 1000 (62) |  |  | |
|  | Squamous Cell | 293 (18) |  |  |  |
|  | Non-small cell, not specified | 115 (7) |  |  |  |
|  | Small Cell | 98 (6) |  |  |  |
|  | Large Cell | 54 (3) |  |  |  |
|  | Other | 54 (3) |  |  |  |
| **Head and Neck Subsites** | Oropharynx |  | 704 (37) |  | |
|  | Lip & Oral Cavity |  | 625 (32) |  |  |
|  | Larynx |  | 426 (22) |  |  |
|  | Unknown |  | 91 (5) |  |  |
|  | Hypopharynx |  | 74 (4) |  | |

** NOS, not otherwise specified*
