# Supplementary material for: Nicotine dependence as a risk factor for upper aerodigestive tract (UADT) cancers: A mediation analysis
Source: PLoS One. 2020 Aug 28;15(8):e0237723. doi: 10.1371/journal.pone.0237723 (PMC7454981; doi:10.1371/journal.pone.0237723)
Supplement: S2 Table — (DOCX) [file pone.0237723.s003.docx]

**S2 Table: Fagerström Dependence Variable Participant Response Rate and Scoring**

| **Fagerström Dependence Variables** | **Category** | **Lung Cases**  **No. (%)** | **HNC Cases**  **No. (%)** | **Controls**  **No. (%)** |
| --- | --- | --- | --- | --- |
| **Total number of participants administered FTND questionnaires** | | 1130 | 680 | 604 |
| **1. How soon after you wake up do you smoke your first cigarette?** | After 60 minutes | 270 (25) | 180 (28) | 355 (62) |
|  | 31-60 minutes | 197 (18) | 102 (16) | 94 (16) |
|  | 6-30 minutes | 356 (33) | 224 (34) | 85 (15) |
|  | Within 5 minutes | 253 (24) | 148 (23) | 39 (7) |
|  | Missing | 54 | 26 | 31 |
| **2. Do you find it difficult to refrain from smoking in places where it is forbidden?** | No | 797 (75) | 534 (80) | 524 (88) |
|  | Yes | 272 (25) | 137 (20) | 72 (12) |
|  | Missing | 61 | 9 | 8 |
| **3. Which cigarette would you hate most to give up?** | Any other | 461 (48) | 270 (47) | 380 (75) |
|  | The first in the morning | 502 (52) | 302 (53) | 126 (25) |
|  | Missing | 167 | 108 | 98 |
|  |  |  |  |  |
| **4. How many cigarettes per day do you smoke^a^?** | 10 or less | 202 (18) | 315 (26) | 279 (47) |
|  | 11-20 | 483 (44) | 590 (48) | 206 (35) |
|  | 21-30 | 293 (27) | 224 (18) | 71 (12) |
|  | 31 or more | 115 (11) | 101 (8) | 38 (6) |
|  | Missing | 37 | 0 | 10 |
|  |  |  |  |  |
| **5. Do you smoke more frequently during the first hours after awakening than during the rest of the day?** | No | 752 (73) | 465 (73) | 542 (92) |
|  | Yes | 282 (27) | 176 (27) | 44 (8) |
|  | Missing | 96 | 39 | 18 |
| **6. Do you smoke even if you are so ill that you are in bed most of the day?** | No | 803 (76) | 524 (82) | 494 (84) |
|  | Yes | 251 (24) | 113 (18) | 93 (16) |
|  | Missing | 76 | 43 | 17 |
| **Modified Fagerström^b^**  **(Item 1 & Item 4)** | Low Dependence |  | 277 (42) | 433 (76) |
|  | High Dependence |  | 377 (58) | 134 (24) |
|  | **Total** |  | ***654*** | ***567*** |
|  |  |  |  |  |
| **Overall Total Fagerström Score**  (Clinical Categories^c^) | 0 to 3 (Low dependence) | 346 (40) | 222 (42) | 360 (75) |
|  | 4 to 6 (Moderate dependence) | 336 (39) | 209 (39) | 83 (17) |
|  | 7 to 10 (High dependence) | 181 (21) | 99 (19) | 37 (8) |
|  | ***Total*** | ***863*** | ***530*** | ***480*** |
| **Overall Completion** | All 6 FTND questions answered | 1873 (78) | | |

^a^ Information of Item 4 was collected from the participants at the study onset, whereas the full FTND questionnaires were started to be administered 2 years after the study onset. ^b^Modified Fagerström was used for the HNC analysis to accommodate the incomplete Fagerström questionnaires. ^c^Clinical Categories: used in nicotine replacement therapies to prescribe dosage of nicotine patches or gum [40].
