# Supplementary material for: Nicotine dependence as a risk factor for upper aerodigestive tract (UADT) cancers: A mediation analysis
Source: PLoS One. 2020 Aug 28;15(8):e0237723. doi: 10.1371/journal.pone.0237723 (PMC7454981; doi:10.1371/journal.pone.0237723)
Supplement: S3 Table — (DOCX) [file pone.0237723.s004.docx]

**S3 Table: The Effect of Nicotine Dependence on the Lung Cancer and Head and Neck cancer risk, mediated through tobacco packyears**

|  |  | **Lung Cancer** | | | | | |  | **Head and Neck Cancer** | | | | | |
| --- | --- | --- | --- | --- | --- | --- | --- | --- | --- | --- | --- | --- | --- | --- |
|  |  | **OR** | **(95%CI)** | **Effect Type** | **Estimate** | **(95%CI)** | **PM** |  | **OR** | **(95%CI)** | **Effect Type** | **Estimate** | **(95%CI)** | **PM** |
| 1. How soon after you wake up do you smoke your first cigarette? | After 60 min | Reference | | Direct | 1.35 | (1.15, 1.58) | 74% |  | Reference | | Direct | 1.30 | (1.10, 1.52) | 67% |
|  | 31-60 min | 1.62 | (1.13, 2.31) |  |  |  |  |  | 1.32 | (0.87, 2.00) |  |  |  |  |
|  | 6-30 min | 2.01 | (1.41, 2.88) | Indirect | 1.40 | (1.26, 1.54) |  |  | 1.86 | (1.25, 2.76) | Indirect | 1.17 | (1.08, 1.26) |  |
|  | Within 5 min | 2.43 | (1.53, 3.84) |  |  |  |  |  | 1.87 | (1.12, 3.11) |  |  |  |  |
| 2. Do you find it difficult to refrain from smoking in places where it is forbidden? | No | Reference | | Direct | 1.61 | (1.07, 2.43) | 62% |  | Reference | | Direct | 0.84 | (0.53, 1.32) | n/a |
|  | Yes | 1.39 | (0.99, 1.97) | Indirect | 1.30 | (1.01, 1.67) |  |  | 0.82 | (0.54, 1.23) | Indirect | 1.41 | (1.06, 1.86) |  |
| 3. Which cigarette would you hate most to give up? | Any Other Time | Reference | | Direct | 1.69 | (1.23, 2.33) | 59% |  | Reference | | Direct | 1.86 | (1.31, 2.63) | 54% |
|  | The first in the morning | 1.80 | (1.35, 2.42) | Indirect | 1.33 | (1.12, 1.59) |  |  | 1.78 | (1.28, 2.49) | Indirect | 1.18 | (0.99, 1.40) |  |
| 5. Do you smoke more frequently during the first hours after awakening than during the rest of the day? | No | Reference | | Direct | 1.67 | (1.10, 2.54) | 60% |  | Reference | | Direct | 2.06 | (1.28, 3.34) | 48% |
|  | Yes | 1.74 | (1.16, 2.60) | Indirect | 1.32 | (1.04, 1.67) |  |  | 1.76 | (1.15, 2.70) | Indirect | 1.07 | (0.85, 1.37) |  |
| 6. Do you smoke even if you are so ill that you are in bed most of the day? | No | Reference | | Direct | 0.77 | (0.52, 1.13) | n/a |  | Reference | | Direct | 0.50 | (0.32, 0.77) | n/a |
|  | Yes | 0.85 | (0.61, 1.19) | Indirect | 1.37 | (1.11, 1.70) |  |  | 0.49 | (0.33, 0.73) | Indirect | 1.42 | (1.11, 1.82) |  |
| **Total Fagerström Score** |  | 1.19 | (1.10, 1.30) | Direct | 1.18 | (1.07, 1.30) | 85% |  | 1.14 | (1.03, 1.25) | Direct | 1.16 | (1.05, 1.29) | 70% |
|  |  |  |  | Indirect | 1.25 | (1.16, 1.34) |  |  |  |  | Indirect | 1.11 | (1.05, 1.18) |  |

Models are adjusted for age, sex, ethnicity, education, smoking status, age started smoking, and family history of lung/HNC cancer. Additionally, for the HNC model, it was also adjusted for average daily alcohol consumption (grams/day). The table demonstrates the odds ratio and 95% confidence interval (OR, 95%CI) and the mediated effects associated with each Fagerström dependence variable (Items 1-3 and 5-6) and for the total Fagerström score (accumulated score from the scores for each of the dependence variables). Because the mediator was packyears, item 4 was excluded from the total Fagerström score. “PM” refers to the proportion mediated through packyears. “n/a” refers to occurrences when the proportion mediated could not be estimated when the direct and indirect effects were in opposite directions.
